# Total and temporal patterning of physical activity in adolescents and associations with mental wellbeing

**DOI:** 10.1186/s12966-023-01553-8

**Published:** 2024-01-08

**Authors:** Abdulwahab D. Alshallal, Olivia Alliott, Soren Brage, Esther M. F. van Sluijs, Paul Wilkinson, Kirsten Corder, Eleanor M. Winpenny

**Affiliations:** 1 MRC Epidemiology Unit, University of Cambridge, Cambridge, UK; 2 Department of Psychiatry, University of Cambridge, Cambridge, UK

**Keywords:** Physical activity pattern, Mental wellbeing, Adolescents

## Abstract

**Background:**

There is limited understanding of the extent to which differences in physical activity across the day and week may be associated with mental wellbeing. Such an understanding is needed for better targeting of interventions. In this study, we describe total and temporal patterning of physical activity across the week in adolescents (age 13-14y) and assess their prospective associations with mental wellbeing.

**Methods:**

1,983 13-14-year-old adolescent participants based in Cambridgeshire and Essex, recruited between 2016 and 2017 into the Get Others Active Trial provided data at baseline and 4 months. Physical activity was measured at baseline using wrist-worn accelerometers across different time segments (whole week, weekday schooltime, weekday out of school, and weekend), and operationalized as average movement-related acceleration for each time segment. Mental Wellbeing at baseline and 4 months was measured using the Warwick Edinburgh MentalWellbeing Scale. Associations between physical activity across different time segments (whole week, weekday schooltime, weekday out of school, and weekend) and mental wellbeing at 4 months were investigated using sex-stratified multi-level regression models, adjusted for covariates, and both adjusted and unadjusted for baseline mental wellbeing.

**Results:**

Our analyses found positive associations between physical activity and mental wellbeing at 4 months, unadjusted for baseline wellbeing. Among girls, positive associations were shown when considering physical activity across the whole week 0.07 (95% CI, 0.03–0.12), and across all separate time periods studied: weekday schooltime 0.07 (95% CI, 0.02–0.11), weekday out-of-school time 0.07 (95% CI, 0.03–0.12), and weekend 0.07 (95% CI, 0.02–0.11). For boys, similar associations were observed for activity across the week 0.07 (95% CI, 0.03–0.11), during weekday schooltime 0.08 (95% CI, 0.04–0.12), and weekday out-of-school time 0.07 (95% CI, 0.03–0.11), but not the weekend 0.01 (95% CI, -0.03-0.05). For both girls and boys, associations were attenuated below significance after adjusting for baseline wellbeing.

**Conclusions:**

This longitudinal analysis showed positive associations between physical activity and later mental wellbeing in both male and female adolescents across most time segments. Higher physical activity throughout the week may be associated with better mental wellbeing in the adolescent population. Further research is required to understand determinants of change in wellbeing over time.

**Trial Registration:**

Registration Number: ISRCTN31583496. Registered: 18/02/2014.

**Supplementary Information:**

The online version contains supplementary material available at 10.1186/s12966-023-01553-8.

## Introduction

Physical activity is essential for healthy growth and physical maturation through childhood and remains important throughout the lifecourse [1]. Adults who are physically active have better health outcomes, mood, self-esteem, and overall mental wellbeing [2, 3]. Adoption of physical activity early in life is associated with higher physical activity and positive mental and physical outcomes later in life [1]. Concerningly, physical activity tends to decrease throughout the life course for most individuals. Based on observed trends worldwide, the sharpest decline happens during adolescence, a phase of life also characterized by significant weight gain [4–7]. The reasons for this decline are still not well understood [8].

Physical activity volume refers to the total amount of physical activity accumulated over a given time period [9]. Patterning of physical activity refers to the temporal structure of physical activity over a specified time period [10]. The total volume of physical activity may be accumulated in different ways across the week in different populations [11]. For example, a cross-sectional study on physical activity patterns of pre-adolescents showed that those classified as highly active had longer bouts of moderate physical activity (MPA) and vigorous physical activity (VPA) before, during, and after school and at lunchtime independent of socioeconomic status (SES), age, gender, and body mass index (BMI), with the greatest differences in physical activity across the study population observed after school [12]. However, the patterning of physical activity across the week may vary with age, and most of the literature exploring change in activity patterns across the week has focused on children and adults rather than adolescents [13, 14].

Adolescence is defined by the World Health Organization as the period of life from the ages of 10 to 19 [15]. A major public health concern worldwide is the mental health of the adolescent population [16, 17]. The 2021 NHS England Mental Health Survey showed that nearly 40% of young people aged 6–16 have exhibited mental health deterioration compared to their 2017 survey results [18]. The prevalence of depression is believed to have significantly increased in this demographic during the COVID-19 pandemic with an estimated prevalence of 25.2% among youth aged 4–18 [19, 20]. Time spent physically active is inversely associated with the incidence of mental health disorders, including depression [21–23].

A similar but seldom studied health indicator in relation to physical activity is mental wellbeing. Mental wellbeing is conceptualized as more than just the absence of mental illness, encompassing positive experience of life, including overall affect, life satisfaction, positive functioning, sense of purpose, among other experiences [24]. The few studies investigating the relationship between physical activity volume and wellbeing have had mixed results [25]. Bell et al. (2019) have previously found no association between accelerometer-assessed physical activity volume and mental wellbeing at three years in adolescents [26]. Conversely, it has been shown that highly active adolescents were more likely to report positive affect and quality of life at 2 years compared to those who were less active [27]. Costigan et al. (2019) also found an inverse U-shape association in this demographic with wellbeing reducing once the total daily volume exceeds 35 min at higher physical activity intensities [28]. The patterning of physical activity and its relation to mental wellbeing in shorter time periods and at different time segments of the week remains unexplored, particularly in the adolescent population.

Previous literature suggests that the relationship between physical activity and mental wellbeing may differ by sex. A positive association has been found between overall weekly physical activity volume and mental wellbeing in 13–15-year-old female but not male adolescents [29]. In contrast, a cross-sectional study of 19–20-year-old college students found that self-reported vigorous physical activity volume was associated with higher positive affect in both males and females [30]. Further research exploring whether sex differences exist for the association between the distribution of physical activity volume and mental wellbeing is warranted [31].

To date, much of the available research examining the association of physical activity volume with mental wellbeing has been conducted on adults or children; studies conducted on adolescents are limited. Moreover, many previous studies are cross-sectional and lacked a temporal component that may be more appropriate for examining this association. Studies investigating physical activity patterning across the week in young people focus simply on in-school versus out-of-school comparison of moderate-to-vigorous physical activity (MVPA) and weekdays vs. weekends [32]. There is limited understanding of the extent to which differences in the patterning of physical activity volume across the day and week may be associated with mental wellbeing. Understanding how physical activity volume is patterned differently in different groups and how these patterns relate to mental wellbeing may provide better targeting of interventions at specific times of the week for high-risk groups.

In response, this study aims to describe the total volume and patterning of physical activity volume across the week among adolescents aged 13–14yearold, and assess prospective associations with mental wellbeing. We will address the following research question: How are total volume and patterning of physical activity volume across the week associated with mental wellbeing at 4-month follow-up?

## Methods

The reporting and analyses in this study follow the Strengthening the Reporting of Observational Studies in Epidemiology (STROBE) guidelines (See Supplementary Material 1) [33].

### Survey design and participants

This study is a secondary analysis of the randomized-controlled trial Get Others Active (GoActive). The GoActive trial was conducted between 2016 and 2018 in schools located in Essex and Cambridgeshire, with the primary aim of increasing MVPA in 13–14-year-old adolescents. The study received ethical approval from the University of Cambridge Psychology Research Ethics Committee (PRE.2015.126). All participants provided written informed assent, and parents provided passive consent [34]. The GoActive trial was not successful in increasing the MVPA of participants. No intervention effects on wellbeing were observed at any time point investigated across the intervention evaluation, thus allowing secondary data analysis across both trial arms [35].

Further details on the results of this trial have previously been reported [34, 36]. Of the 2,862 13-14-year-old adolescents who provided consent, 2,638 (92%) provided data on accelerometer-measured physical activity at baseline. All participants included in the analysis have valid accelerometer-measured physical activity data across the week. At age 13–14 years, a time of rapid adolescent development, physical activity and mental wellbeing are likely to change over time. Based on previous research, we expect the influence of physical activity on mental wellbeing to take place across a relatively short time scale [37, 38]. In the present analysis, we, therefore, used data collected at baseline and the first assessment after baseline, which was at mid-intervention (4 months from baseline), to examine associations between physical activity and wellbeing.

### Measures

#### Physical activity

Participants were asked to wear a triaxial accelerometer (AX3, Axivity) continuously for seven days on their non-dominant wrist to assess baseline physical activity. Raw measured acceleration was calibrated to local gravity, and movement-related acceleration was calculated using the Euclidian Norm Minus One (ENMO) as previously described [39, 40]. Non-wear periods were identified as stationary time segments lasting over 60 min. The wrist accelerometry method has been validated to assess physical activity and also provides more complete data records than commonly used hip-worn monitors among adolescents [41–43]. To investigate physical activity volume across different segments of the day, ENMO was averaged across the whole week as well as for three separate time segments (weekday schooltime: 9 AM-3 PM, weekday out-of-school: 6 AM–9 AM, and 3 PM-12 AM, weekend: 6 AM-12 AM across both weekend days) [44]. Time (minutes/day) spent in MVPA for each time segment was estimated using a cut point equivalent to 2000 Actigraph counts per minute [34].

#### Mental wellbeing

Participant mental wellbeing was measured at baseline and at 4 months via the Warwick Edinburgh Mental Wellbeing Scale. The scale consists of 14 items that ask students what best describes their experiences of a collection of statements over the past 2 weeks. Examples include “I’ve been feeling optimistic about the future,” “I’ve been feeling close to other people”, and “I’ve been dealing with problems well.” Questions relate to positive affect, relationships, and emotional functioning. Each item is rated on a five-point Likert scale with responses “none of the time,” “rarely”, “some of the time,” “often” and “all of the time” scored 1–5, respectively [34].

The Warwick Edinburgh Mental Wellbeing Scale, used to measure mental wellbeing in this study was developed in the UK and has previously shown high internal consistency and construct validity for adolescents in Norway [45]. It has also been shown to have cross-cultural validity in a sample of Brazilian adolescents, suggesting the applicability of the measure for adolescents of multiple ethnic demographics [46].

#### Covariates

At baseline, 13-14-year-old adolescents completed questionnaires to measure variables of interest, including age, sex, and ethnicity, and to report average sleep and wake time on weekdays and weekends. Students also responded to questions related to the family affluence scale (FAS) as a measure of socioeconomic status [47]. Anthropometric measurements, including body fat% (via bioelectric impedance), were taken by trained research staff. Time spent sleeping was measured by calculating the difference between self-reported sleep and wake times during term time. Participants were asked “Please tell us what time you usually wake up and go to sleep (not to bed) during term time.” Further details on these measurements from the GoActive trial have previously been published [35]. A Directed Acyclic Graph (DAG) was used to identify possible confounders. Age, sex, sleep, ethnicity, body fat%, and FAS score have all been cited previously in the literature as potential individual-level confounders of the association between physical activity and mental wellbeing at follow-up (See Supplementary Material 2). All models were also adjusted for the school-level covariates: school socioeconomic status (percentage of pupils receiving pupil premium) and trial group. Previous literature, as well as exploratory plots of our data suggested sex differences in the relationship between physical activity at baseline and mental wellbeing; thus, analyses were stratified by sex, and the remaining covariates were included in the regression model [48].

### Statistical analysis

#### Descriptive statistics

Participants with complete data on all variables were included in the analysis. Chi-squared tests and t-tests were used as appropriate to compare included to excluded participants. Mean and standard deviation (SD) were used to describe continuous variables. Categorical variables were summarized via numbers and percentages. Participants of non-white ethnicities were combined in one variable due to their small representations in the sample. Paired t-tests were used to explore differences in physical activity during different time segments and mental wellbeing between boys and girls. Paired t-tests were also used to examine differences between different time-segments of physical activity at baseline. Spearman’s correlation coefficients were calculated to assess relationships between baseline mental wellbeing and mental wellbeing mid-intervention.

#### Regression analysis

Mean mental wellbeing score at mid-intervention was regressed on overall physical activity volume (mean ENMO) at baseline using a multilevel model which adjusted for potential confounders and clustering of individuals within schools. We conducted separate regression models to investigate physical activity averaged across the whole week and in different time-segments (weekday schooltime, weekday out of school, weekend). A random intercept term was added for school ID to consider the clustering of individuals within schools. Random effects were added using the “lme4” package in R. Trial group, and school SES were also included given the multi-school trial design and as potential school-level confounders.

The main model was built by adopting a sequential nesting procedure for hierarchical regression, wherein individual-level covariates were added one at a time before adding school-level covariates. Likelihood ratio tests were used to assess improved model fit after each addition of a covariate. R^2^ and ICC values were also reported.

The first model was unadjusted for confounders. The second model, our main model, assessed the prospective association of baseline physical activity with mental wellbeing at 4 months adjusted for confounders. In the third model, we added adjustment for baseline mental wellbeing to assess the association of baseline physical activity with change in mental wellbeing over the 4-month period.

## Results

### Descriptive statistics

Of the 2,638 GoActive participants that provided accelerometer-measured physical activity data, 210 (8%) participants had missing wellbeing data at 4-month follow-up, and a further 445 (17%) participants had missing data on covariates. The final analysis used information from 1,983 (75% of the initial sample) participants who had data on all variables. Excluded participants had lower mental wellbeing at baseline and 4 months (p < 0.05) and were more likely to be male (55% compared to 49% in the final sample). Ethnic proportions, sex-specific body fat%, sleep, and socioeconomic status were not significantly different from individuals included in the analysis.

Chi-squared tests suggest a small but significant decline in mental wellbeing at baseline and at 4 months (p < 0.0001) in both boys and girls (Table 1). However, there was no significant difference in the change in mental wellbeing between the sexes. There was also a moderate positive correlation between baseline and mental wellbeing at 4 months (r = 0.59, p < 0.0001). Table 1 shows baseline measurements of participants’ physical activity based on average hourly ENMO at baseline. Time in MVPA was also reported for comparison. Boys were more active than girls overall. Physical activity volume is highest during school time and lowest during the weekends for all demographics. There was a significant difference between average ENMO between school time and weekend (p < 0.0001) as well as between out-of-school and weekend ENMO (p < 0.0001).

**Table 1:** Participant Characteristics based on questionnaire and anthropometry data collected at baseline

	Overall	Boys	Girls
Number of Participants	1983	979 (49.4%)	1004 (50.6%)
Age in years, mean (SD)	13.75 (0.30)	13.75 (0.31)	13.75 (0.30)
White, n (%)	1723 (86.9%)	841 (85.9%)	882 (87.8%)
Body Fat % (IQR)	20.7 (12.8–28.7)	13.2 (9.9–18.6)	27.2 (21.9–32.8) ***
Average Sleep, mean (SD)	9.34 (1.18)	9.30 (1.22)	9.38 (1.14)
**Family Affluence**			
Low Affluence, n (%)	256 (12.9%)	119 (12.2%)	137 (13.6%)
Medium Affluence, n (%)	843 (42.5%)	391 (39.9%)	452 (45.0%) **
High Affluence, n (%)	884 (44.6%)	469 (47.9%)	415 (41.3%) **
**Physical Activity based on ENMO (milli-g)**			
Mean (Whole Week) (SD)	42.70 (12.56)	46.43 (13.85)	39.06 (9.90) ***
Weekday Schooltime (SD)	45.16 (13.33)	49.13 (14.52)	41.28 (10.73) ***
Weekday Out of School (SD)	30.61 (10.13)	31.86 (11.47)	29.39 (8.45) ***
Weekend (SD)	35.14 (18.14)	37.59 (21.85)	32.75 (13.17) ***
**Physical Activity based on MVPA (min)**			
Mean (Whole Week) (SD)	36.16 (18.53)	42.17 (19.76)	30.30 (15.11) ***
Weekday Schooltime (SD)	13.24 (6.97)	15.47 (7.43)	11.05 (5.71) ***
Weekday After School (SD)	18.63 (12.13)	20.96 (13.37)	16.35 (10.30) ***
Weekend (SD)	28.87 (24.65)	33.74 (29.14)	24.12 (18.09) ***
**Mental Wellbeing Score**			
Baseline, mean (SD)	3.50 (0.66)	3.63 (0.63)	3.38 (0.68)
Mid-Intervention, mean (SD)	3.45 (0.72)	3.59 (0.68)	3.31 (0.73)

Baseline and Mid-Intervention participant wellbeing scores are the mean score of the 14-item Warwick Edinburgh Mental Wellbeing Scale

Hours between 9 AM and 3 PM were classified as schooltime. Hours between 6 AM to 9 AM and 3 PM to 12 AM were classified as out-of-school time

MVPA values are based on the average minutes per time segment

**Significant difference between boys and girls p ≤ 0.001

***Significant difference between boys and girls p ≤ 0.0001

### Associations of PA with mental wellbeing

Sex-stratified regression model results are shown in Table 2. Model 1 shows the unadjusted association of physical activity volume with mental wellbeing at 4 months. Model 2, the main model, shows the results of the prospective association of physical activity volume with mental wellbeing at 4 months, adjusted for confounders. For Model 2, small positive associations were found for both sexes across all time segments except for weekend physical activity in boys. Model 3 shows the results of the prospective association of physical activity volume with change in mental wellbeing, adjusted for confounders. No significant associations were found for model 3.

**Table 2:** Associations between average physical activity at baseline with mental wellbeing at 4 months

	Model 1	Model 2	Model 3
Exposure (Boys)			
Mean (Whole Week) (SD)	**0.07 (0.02, 0.11)**	**0.07 (0.03, 0.11)**	0.02 (-0.02, 0.05)
Mean Weekday Schooltime (SD)	**0.08 (0.04, 0.12)**	**0.08 (0.04, 0.12)**	0.03 (-0.01, 0.07)
Mean Weekday Out of School (SD)	**0.07 (0.03, 0.11)**	**0.07 (0.03, 0.11)**	0.02 (-0.02, 0.06)
Mean Weekend (SD)	0.01 (-0.03, 0.05)	0.01 (-0.03, 0.05)	-0.02 (-0.05, 0.02)
**Exposure (Girls)**			
Mean (Whole Week) (SD)	**0.09 (0.04, 0.13)**	**0.07 (0.03, 0.12)**	0.02 (-0.02, 0.06)
Mean Weekday Schooltime (SD)	**0.08 (0.03, 0.12)**	**0.07 (0.02, 0.11)**	0.02 (-0.02, 0.05)
Mean Weekday After School (SD)	**0.09 (0.04, 0.13)**	**0.07 (0.03, 0.12)**	0.01 (-0.02, 0.05)
Mean Weekend (SD)	**0.08 (0.04, 0.12)**	**0.07 (0.02, 0.11)**	0.03 (-0.01, 0.06)

Mental Wellbeing Score Range is 0–5. Regression coefficients are based on one SD of ENMO

All models restricted to individuals with complete data on all covariates. Covariates are ethnicity, age, SES, school SES, sleep, body fat%, and trial group

All models account for clustering by school

Model 1: Unadjusted for all covariates

Model 2: Adjusted for all covariates except baseline mental wellbeing

Model 3: Adjusted for all covariates including baseline mental wellbeing

Significant associations (p < 0.05) are shown in bold

## Discussion

### Summary of main findings

The purpose of this study was to investigate associations between physical activity in different time segments with mental wellbeing in 13-14-year-old adolescents. Multivariable models investigating the prospective association showed a statistically significant positive association between physical activity volume and mental wellbeing at 4 month follow-up, but the effect sizes are small. Results show that on average, for every SD higher baseline ENMO, there is a 10th of a SD higher mental wellbeing at 4 months. When physical activity volume is separated by time period, associations with wellbeing in boys were seen for weekday school and out-of-school physical activity volume, but not for weekend physical activity volume. In contrast, associations in girls were seen across all time segments. No association was found when adjusting for baseline mental wellbeing, suggesting physical activity volume may not influence change in mental wellbeing over time, and also raising the possibility that associations may be driven by reverse causation.

### Comparison with previous research

Our findings on the overall association of higher volume of physical activity with higher mental wellbeing are in agreement with previous literature on the associations between physical activity among adolescents and reductions in mental illness such as depression [22]. Moreover, our findings align with Molcho et al. (2021) who found positive cross-sectional associations between physical activity in adolescents and measures of wellbeing [49]. With the exception of weekend physical activity volume in boys, we found that a higher volume of physical activity is positively associated with mental wellbeing across all time segments of the day and week investigated in our study. To our knowledge, this is the first prospective study in adolescents that has investigated the association of physical activity timing across the week with mental wellbeing [50–52].

However, similar to previous cross-sectional studies, our findings here cannot rule out the possibility of reverse causation. Although we found a prospective association between physical activity volume and wellbeing at 4-month follow-up, this association was no longer significant after control for baseline mental wellbeing. Since mental wellbeing at baseline and 4-month follow-up shows a moderate correlation (r = 0.59) it may be that the prospective association seen is in fact, driven by a cross-sectional association between physical activity and wellbeing at baseline, which could reflect causal effects in either direction.

Previous observational studies assessing associations of physical activity with depressive symptoms have found non-significant or significant but smaller effect sizes in longitudinal studies compared to cross-sectional studies suggesting some reverse causality [23, 53]. The cross-sectional study by Costigan et al. (2019), similar to our analysis, found a positive association in both sexes but only for positive affect and vigorous-intensity physical activity [28]. Longitudinally, however, the relationship is unclear. Barth Vedoy et al. (2021) found a positive association only in girls for total physical activity volume with mental wellbeing at 3 years [29]. In contrast, Bell et al. (2019) found no association in either sex over a 3-year period [26]. Being highly active has also been found to reduce chances of lower mental wellbeing at 2 years follow-up [27]. In comparison to existing literature, our findings suggest that apart from weekend physical activity in boys, there is no variation with time segment of the day for this pathway.

The lack of an association between physical activity volume and mental wellbeing seen in boys at the weekend is an interesting and novel finding. This may be explained by the nature of the physical activity in which boys participate at the weekend, which may include sports participation and competition. Among British boys aged 9–16, recreational football accounted for 60% of weekend physical activity [54]. A key feature of organized sports is a higher proportion of activity spent at a vigorous intensity, specifically for boys. Prior studies on 9-11-year-old children found a significant difference in VPA on weekends and weekdays between boys and girls, with boys also engaging in more frequent and longer bouts of VPA relative to light physical activity than girls on weekends [55, 56]. There may be an optimal amount of physical activity accumulated at different intensities and more time spent at lower physical activity intensities may be beneficial for mental wellbeing. Further analyses of other reasons for the differences in associations seen across different time segments in boys would be an important extension of this work. This would require a more detailed analysis of the ways in which different kinds of physical activity are related to wellbeing, for example exploring factors such as domain, social setting, competitiveness of physical activity engagement, and potential mediators of the link with wellbeing.

### Strengths and limitations

The GoActive study included a large sample, representative of the East of England, and our findings are, therefore, most relevant to this region and other high-income settings [52]. Although the ethnic diversity of the participants in GoActive was similar to that of England and Wales, due to their lower representation in the GoActive study, this analysis may not accurately describe the physical activity behavior of 13-14-year-old adolescents from ethnic minorities and lower SEP groups who may experience more barriers to physical activity [35, 52]. Since the GoActive study is a trial, it is possible that the intervention may have influenced the associations examined in this cohort analysis. However, since our exposure here is at baseline, prior to the randomization of study sites, and no intervention effects on neither physical activity nor wellbeing were observed in this trial, we do not think there is any likelihood of bias of the effect estimates of our analysis. In addition, we adjusted for trial arm in our analyses, and the coefficient for this term was small and non-significant.

The use of accelerometry to measure physical activity in this study overcomes the potential biases of self-reported physical activity, including social desirability and recall bias. In addition, although mental wellbeing cannot be objectively assessed, we used a well-validated instrument to assess mental wellbeing, the WEMWBS [57]. We were able to account for a wide range of confounders that may influence both physical activity and mental wellbeing; however, there may be some residual confounding. For example, one confounder that may drive both physical activity and mental wellbeing is the strength of friendship networks, which could not be accounted for [58]. Although we observed prospective observations between physical activity volume and mental wellbeing, this may be due to reverse causation if mental wellbeing tracks over a period longer than the 4-month study period. This analysis cannot rule out reverse causation for the associations seen since adjusting for baseline mental wellbeing attenuated associations below significance. Although we looked at an association over a 4-month study period, different associations may have been seen at shorter or longer follow-ups. Similarly, this analysis focused on adolescents at age 13–14 years. Because 13-14-year-old adolescents may not be representative of all adolescents, different associations may be expected at different ages during adolescence.

## Conclusions

We found positive associations between physical activity volume and mental wellbeing 4 months later among 13-14-year-old adolescents. Associations were similar for girls irrespective of when in the week physical activity occurred but for boys associations were stronger for weekday than weekend physical activity. Physical activity was not associated with change in wellbeing. Further research is necessary to establish if increasing physical activity volume would cause improved mental wellbeing or if improved wellbeing would lead to higher activity levels, or both. Meanwhile, these findings support an increased emphasis on physical activity within the school day to promote mental wellbeing.

### Electronic supplementary material

Below is the link to the electronic supplementary material.

Supplementary Material 1

Supplementary Material 2

## Data Availability

The datasets generated and/or analysed during the current study cannot be made openly available because of ethical and legal considerations. Non-identifiable data can be made available to bona-fide researchers on submission of a reasonable request to datasharing@mrc-epid.cam.ac.uk. The principles and processes for accessing and sharing data are outlined in the MRC Epidemiology Unit Data Access & Data Sharing Policy.

## List of abbreviations

ENMO: Euclidean Norm Minus One
GoActive: Get Others Active
VPA: Vigorous Physical Activity
MVPA: Moderate to Vigorous Physical Activity
WEMWBS: Warwick Edinburgh Mental Wellbeing Scale
