# Supplementary material for: Total and temporal patterning of physical activity in adolescents and associations with mental wellbeing
Source: Int J Behav Nutr Phys Act. 2024 Jan 8;21:5. doi: 10.1186/s12966-023-01553-8 (PMC10775671; doi:10.1186/s12966-023-01553-8)
Supplement: Supplementary file 2 — Supplementary Material 2 [file 12966_2023_1553_MOESM2_ESM.docx]

# Supplementary Material

**Additional File 2.** Directed Acyclic Graph for regression covariates, showing the relationship between the exposure (Physical Activity ENMO) and outcome (Mental Well-Being Mid-Intervention) as well as possible confounders. Black lines denote relationship from covariate to physical activity. Red lines denote relationship between covariate and mental well-being. Yellow lines denote effect of a covariate on body fat %.

***
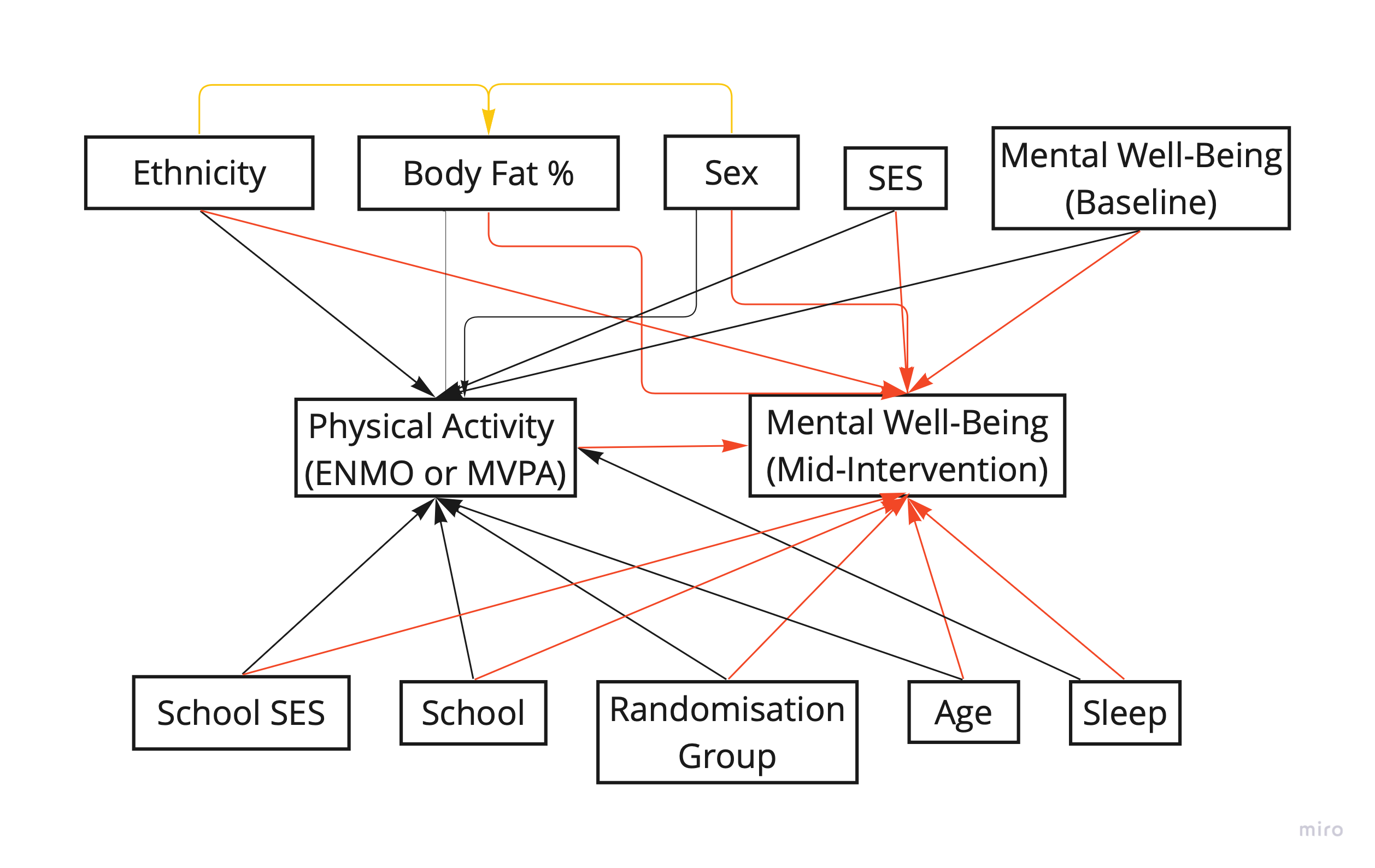
***
